# Giant intradiploic epidermoid cyst with large osteolytic lesions of the skull: a case report

**DOI:** 10.1186/1752-1947-6-85

**Published:** 2012-03-22

**Authors:** Wolfgang Krupp, Alexander Heckert, Heidrun Holland, Jürgen Meixensberger, Dominik Fritzsch

**Affiliations:** 1 University Clinic Leipzig, Department of Neurosurgery, Liebigstraße 20 in 04103 Leipzig, Germany; 2 Evangelical Clinic Oldenburg, Department of Neurosurgery, Steinweg 13-17 in 26122 Oldenburg, Germany; 3 University Clinic Leipzig, Department of Neuroradiology, Liebigstraße 20 in 04103 Leipzig, Germany; 4 University of Leipzig, Translational Centre for Regenerative Medicine, and Faculty of Medicine, Leipzig, Germany

## Abstract

**Introduction:**

We report a case of tumor growth over a period of four decades, presenting with large multicentric lytic lesions of the skull and a profound mass effect, without neurological deficits. Clinical and radiological features of a patient with a giant intradiploic epidermoid and its impact on the choice of treatments are discussed.

**Case presentation:**

An 81-year-old Caucasian man, who had first noticed a painless subcutaneous swelling over the left frontal scalp about 40 years ago, presented after a short episode of dizziness, which he experienced after treatment of focal retinal detachment. Computed tomography (CT) and magnetic resonance imaging (MRI) examinations revealed an exceptionally large tumor involving major parts of the skull with extensive destruction of the bone and distinct deformation of the brain. Considering his age and the absence of neurological deficits or pain, the patient refused the option of tumor removal and cranioplasty, yet agreed to a biopsy, which confirmed the suspected diagnosis.

**Conclusions:**

The course of the disease demonstrates that even patients with large tumors, inducing distinct pathomorphological changes, do not necessarily experience significant impairment of their quality of life without surgery. This is an impressive example of the chance to lead a long and satisfying life without specific medical treatment, avoiding the inherent risks of these procedures. Yet, there is a clear indication for surgery of intradiploic epidermoids in most cases described in the literature.

## Introduction

We report the case of a patient with a progressive swelling of the cranial vault and growing lesions of the skull who refused any further diagnostic procedures over a period of 40 years, although it was urgently recommended by his physicians. When he finally agreed to undergo a magnetic resonance imaging (MRI) investigation, large polycystic calvarial defects and extensive deformation of major parts of the brain were detected.

The patient had an exceptionally large intradiploic epidermoid cyst with extensive destruction of large areas of the skull and distinct deformations of the brain without neurological deficits.

Cranial epidermoid cysts are rare lesions, representing between 0.2% and 1% of all intracranial tumors. Intradiploic epdermoid cysts account for about 25% of these lesions [1-3]. Most of them are slow growing, benign, supposedly congenital tumors which derive from ectodermal remnants misplaced during embryogenesis [4]. A posttraumatic etiology was considered in a few cases [2]. Malignant changes may occur rarely [5].

## Case Presentation

An 81-year-old Caucasian man presented with a large painless swelling of the cranial vault, covering almost the whole left side and also major parts of the right side of the skull.

He had first noticed a small swelling over the left frontal region about 40 years ago. During the following four decades the swelling slowly expanded. For more than 15 years his family physician had recommended an MRI for further examination. The patient refused it over a long time period because he felt no pain or other discomfort. He experienced no significant problems during his customary life activities.

He finally agreed to further diagnostic procedures after a short period of dizziness following an ambulatory treatment of a focal retinal detachment. After this episode no further clinical symptoms appeared. Neurological examination was entirely normal. No cognitive deficits were found.

Examination of the lesion revealed a subcutaneous mass covering most of the left cranial vault and major parts of the right side. Palpation revealed a soft elastic mass, adherent to the surface, without fluctuation.

In contrast to the giant size of the cyst, on first sight no severe deformation of the head had developed. The overlying skin did not show pathologic changes, such as defects or inflammatory signs.

**Figure 1:**
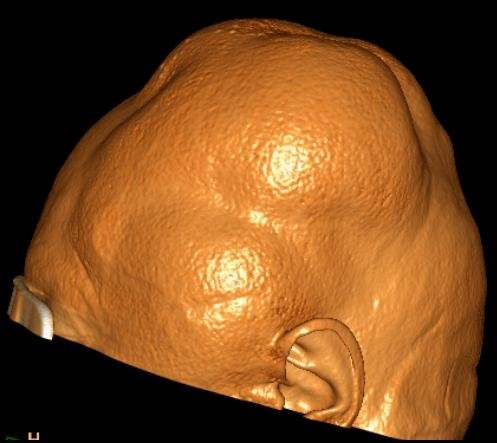
**Spiral computed tomography (CT) scan of the brain with surface volume rendering technique illustrates the bulging of large parts of the scalp**.

**Figure 2:**
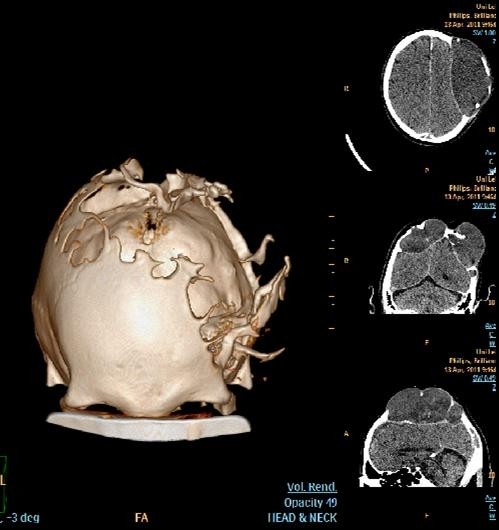
**Volume rendering technique of the computed tomography (CT) scan in the bone intensity window and insets of axial, coronal, and sagittal computed tomography (CT) scans, show the calvarial defects, especially on the left side**.

**Figure 3:**
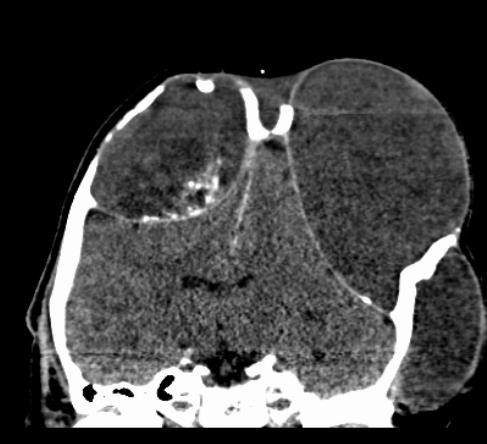
**Coronal reconstruction of computed tomography (CT) scan reveals the lack of the outer table and the thinning of the inner table due to the expansive intradiploic cystic mass**. Intratumoral calcification is shown on the right side.

A spiral computed tomography (CT) (Philips Brilliance 64 CT-scanner) with surface volume rendering technique revealed a large swelling of the scalp, especially over the left hemisphere (Figure 1). Computed tomography (CT) with surface reconstructing and insets of axial, coronal, and sagittal scans demonstrated a giant cystoid lesion of the skull, with large calvarial defects on both sides, left more than right. The outer and the inner table of the skull bone were widely destroyed; in some areas the inner table was thinned out (Figure 2). A distinct compression of both brain hemispheres was caused by the tumor, with a slight midline shift to the right side (Figure 3).

**Figure 4a/b:**
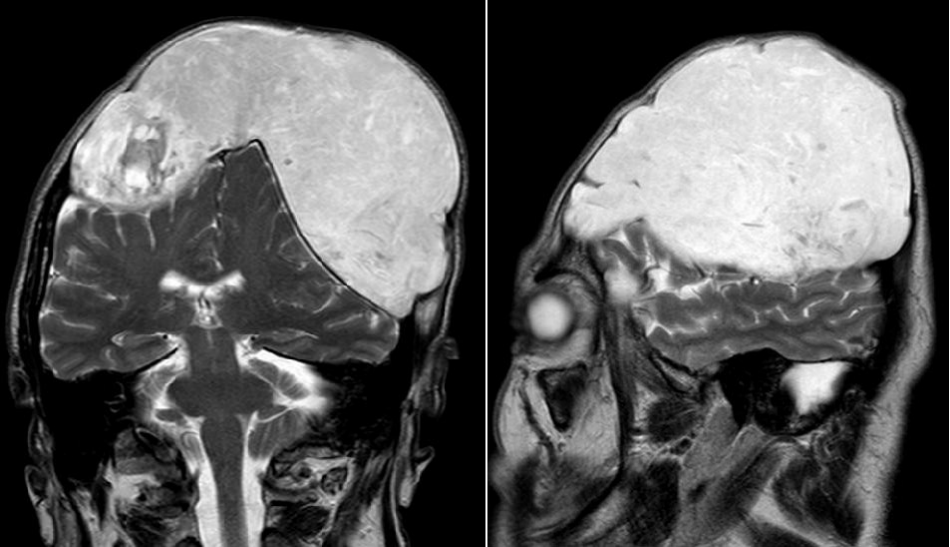
**Magnetic resonance imaging T2 weighted images show the distinct compression of both brain hemispheres and ventricles due to the tumor, measuring 15 × 12 × 10 cm**. An inhomogeneous high signal is shown within the tumor.

**Figure 5:**
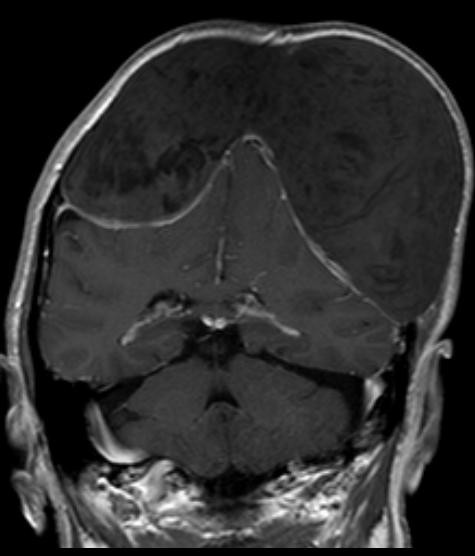
**Magnetic resonance imaging T1 weighted image past gadolinium shows a thickening of the dura, but no enhancement within the epidermoid tumor**.

The MRI scan T2 weighted images (Philips Achieva 1.5 T) revealed an inhomogeneous, mainly hyperintense mass without penetration of the dura. Significant compression of brain hemispheres and ventricles without cerebral edema suggested a slow growing tumor. In diffusion weighted images there was a restriction of diffusion with low signal in the ADC maps (Figure 4a/b). The contrast enhanced T1 weighted images (0.1 mmol/kg Gadovist (gadobutrolum), Bayer Health Care) showed a mild thickening of the intact dura. No enhancement in the epidermoid tumor was found (Figure 5).

In summary, radiological features were suggestive of the presence of an extradural intradiploic epidermoid cyst.

After thorough discussion of the diagnostic findings with the patient, he favored a biopsy to assure the histological classification of the tumor but refused the option of a complete removal of the tumor and subsequent cranioplasty. A biopsy was taken in the left frontal region. Histological examination revealed laminated keratin material, cholesterol crystals, and cellular debris. Pathological findings were consistent with the diagnosis of an epidermoid cyst. Due to a superficial wound infection surgical revision was necessary. His postoperative course was uneventful.

## Discussion

Cushing first described an intradiploic epidermoid cyst in 1922 [6]. A total of 223 cases were reported in the literature by 1990 [7].

Clinically intradiploic epidermoid cysts commonly present as slowly growing, painless lumps in any part of the scalp. Differential diagnosis of these tumors presenting with lytic bone defects includes dermoid cyst, hemangioma, eosinophilic granuloma, fibrous dysplasia, especially with widening of diploic spaces, and several others [8].

In most cases a tentative diagnosis can be made based on radiological features and history of clinical symptoms but definitive diagnosis requires tissue histomorphology [4].

Extradural epidermoids account for about 25% of cranial epidermoids and are located on the scalp, cranial vault or skull base [1]. The cause of the bony defect is hypothesized to be persistent expansive pressure [1].

To the best of our knowledge no intradiploic epidermoid of this large size with extensive bony defects and massive deformation of major parts of the brain has been described in the literature. The time interval between first detection of a subcutaneous swelling by the patient and diagnosis, covering a period of about four decades, also is uncommon, yet corresponds to one other case report, in which a slowly progressive, painless proptosis of an eye has been described [9].

The patient experienced no restrictions of his daily life activities or social contacts. According to the patient the subcutaneous swelling only rarely has been addressed. The patient never felt handicapped by the lesion. Although the tumor did not penetrate the dura, CT and MRI examinations revealed a profound mass effect with an extensive deformation of the brain. In spite of additional large skull defects a jolting of the head or rapid movements did not cause headache or other symptoms. The patient also experienced no problems laying down his head on the left or right side for sleep.

Taking into consideration the age of the patient, his obviously good quality of life, and the risks of a total removal of this giant tumor with subsequent cranioplasty, in our opinion there was no indication for surgery in this case, even if the patient would have agreed to it.

The origin of these tumors is still debated, yet disontogenetic etiology is widely accepted, assuming that the lesion derives from inclusions of ectodermal remnants during closure of the neural tube [2]. In a few cases an acquired origin has been considered, caused by implantation of epidermal fragments in connective tissue [2,3]. In our case there has been no history of head trauma, except for a possible lesion caused by a forceps delivery.

## Conclusions

As evident in our case even giant extradural epidermoids with profound deformation of the brain and extensive lytic skull lesions may allow a normal life without any significant restrictions. The course of the disease, covering a period of more than forty years, also demonstrates that an individual risk-benefit calculation is mandatory in the discussion of a specific treatment, which probably also applies to other diseases. There is no evidence that removal of the tumor within the described time interval would have achieved a positive impact on the life of the patient in spite of the distinct anatomical alterations. Notwithstanding, in most cases surgery is clearly indicated due to clinical or pathomorphological features.

## Consent

Written informed consent was obtained from the patient for publication of this case report and any accompanying images. A copy of the written consent is available for review by the Editor-in-Chief of this journal.

## Competing interests

The authors declare that they have no competing interests

## Authors' contributions

WK examined the patient, interpreted the findings and performed the surgery, and was a major contributor in writing the manuscript. AH examined the patient, interpreted the findings, designed and reviewed the manuscript. HH designed and reviewed the manuscript. JM designed and reviewed the manuscript. DF analyzed and interpreted the radiologic examination findings, and was a major contributor in writing the manuscript. All authors read and approved the final manuscript.

## References

[B1] Cho JH Jung TY Kim IY Jung S Kang SS Kim SH A giant intradiploic epidermoid cyst with perforation of the dura and brain parenchymal involvement Clin Neurol Neurosurg 2007 109 368 373 10.1016/j.clineuro.2006.12.011 17254702

[B2] Locatelli M Alimehmeti R Rampini P Prada F Intradiploic frontal epidermoid cyst in a patient with repeated head injuries: is there a causative relationship? Acta Neurochir (Wien) 2006 148 1107 1110 10.1007/s00701-006-0867-5 16944055

[B3] Enchev Y Kamenov B William A Karakostov V Posttraumatic giant extradural intradiploic epidermoid cysts of posterior cranial fossa: case report and review of the literature J Korean Neurosurg Soc 2011 49 53 57 10.3340/jkns.2011.49.1.53 21494364 PMC3070896

[B4] Kalgutkar A Kini S Jambhekar N Das S Intradiploic primary epithelial inclusion cyst of the skull Ann Diagn Pathol 2006 10 20 23 10.1016/j.anndiagpath.2005.07.007 16414540

[B5] Hoeffel C Heldt N Chelle C Claudon M Hoeffel JC Malignant change in an intradiploic epidermoid cyst Acta Neurol Belg 1997 97 45 49 9107345

[B6] Cushing H A large epidermal cholesteatoma of the parietotemporal region deforming the left hemipshere without cerebral symptoms Surg Gynecol Obstet 1922 34 557 566

[B7] Ciappetta P Artico M Salvati M Raco A Gagliardi FM Intradiploic epidermoid cysts of the skull: report of 10 cases and review of the literature Acta Neurochir (Wien) 1990 102 33 37 10.1007/BF01402183 2407051

[B8] Narlawar RS Nagar A Hira P Raut AA Intradiploic epidermoid cyst J Postgrad Med 2002 48 213 214 12432201

[B9] Sargent EW Garcia P Paniello RC Spector GJ Giant intradiploic epidermoid cyst of greater sphenoid wing causing unilateral proptosis and optic nerve compression Skull Base Surg 1993 3 55 59 10.1055/s-2008-1060565 17170890 PMC1656414

